# Biomechanical assessment of the paediatric foot: using the current evidence

**DOI:** 10.1186/1757-1146-5-S1-P7

**Published:** 2012-04-10

**Authors:** Angela M Evans, Keith Rome

**Affiliations:** 1 Health & Rehabilitation Research Institute, AUT University, Auckland, 92006, New Zealand; 2 University of South Australia, Adelaide, 5000, Australia

## Background

The paediatric flat foot is a frequent presentation in clinical practice, a common concern to parents and continues to be debated. As an entity, it is confused by varied classifications, the notion of well-intended prevention and unsubstantiated, if common, treatment [1]. The paediatric flat foot proforma (p-FFP) is a standardized framework from which to evaluate the paediatric flat foot [2].

## Materials and methods

An algorithm, extending the p-FFP, has been developed to direct assessment and management of the paediatric flatfoot. Based upon best available evidence, this model includes joint hypermobility, body weight and gender as relevant items to assess [3]. The normative data sets using the foot posture index are included and recent reliability studies [4] have identified the value of the ankle lunge test, Beighton scale and the lower limb assessment score in evaluation joint range, hypermobility and quality of life (Table 1).

**Table 1:** Inter-rater reliability: mean inter-rater ICC’s (95% CI’s) and SEM in children aged seven to 15 years (n=30)

Variable	ICC (95% CI)	Mean (SD)	SEM
Foot Posture Index	0.79 (0.38-0.94)	4.3 (2.7)	1.3
Lunge Test	0.83 (0.56-0.94)	43.7 (5.0)	2.9 deg
Beighton Scale	0.73 (0.42-0.88)	2.4 (1.2)	1.2
Lower Limb Assessment Score	0.78 (0.41-0.93)	9.7 (3.3)	2.5

## Results

A recent critical literature review has identified that the resting calcaneal stance position (RCSP), navicular height and Foot Posture Index (FPI-6) are the only three reliable measures of static foot posture [5].

## Conclusions

Further research is required to establish a universal method of assessment of paediatric foot posture. The relevance of static foot posture to pain and shod gait function remains largely unsubstantiated in children, and warrants further investigation.
